# Simulation data for engineering graphene quantum dot epoxy nanocomposites using molecular dynamics

**DOI:** 10.1016/j.dib.2024.110169

**Published:** 2024-02-12

**Authors:** Prathamesh P. Deshpande, Ozgur Keles

**Affiliations:** San Jose State University, 1 Washington Sq., San Jose, CA 95192, USA

**Keywords:** Molecular dynamics, Graphene quantum dots, Epoxy nanocomposite, Chemical functionalization, Mechanical properties

## Abstract

Graphene quantum dots (GQDs) were reported to fill the role of nanofillers that enhance composite properties. Detailed investigation of this nanofiller in composites is largely unexplored. To understand the fundamental mechanisms in play, this study uses molecular dynamics simulations to reveal the effects of GQDs on epoxy properties. Mechanical simulations were performed on three varying GQD chemistries which included a pristine GQD and 2 edge aminated GQDs with different degrees of functionalization (5.2 % and 7.6 %). These GQDs were separately inserted in a polymer matrix across five individual replicates. The nanocomposite mechanical properties were computed using uniaxial strain simulations to display the effect of embedded GQDs.

Specifications Table

**Table:** 

Subject	Computational Mechanics, Computational Materials, Material Science
Specific subject area	Molecular Dynamics simulation of GQD-epoxy nanocomposites
Data format	Raw: LAMMPS trajectory, input scripts, and log files Analysed: stress-strain plots
Type of data	LAMMPS simulation and mechanical property plots
Data collection	Molecular dynamics simulations were performed on a computing cluster at San Jose State University using LAMMPS open-source software. The data was post-processed using custom python scripts to generate the mechanical property predictions.
Data source location	San Jose State University, San Jose, CA 95192
Data accessibility	Repository name: Mendeley Data Data identification number: 10.17632/gmm7rfrsdt.1 Direct URL to data: https://data.mendeley.com/datasets/gmm7rfrsdt/1

## 1 Value of the Data

• The detailed simulation data provides structural evolution of the GQD-epoxy nanocomposite during mechanical deformation. Along with the atom trajectories, the stress-strain response is supplied to link the structure and the properties of nanocomposite models.
• Polymer scientists invested in GQD filled composites can gain substantial insight into the molecular behavior of the material. Also, other computational researchers benefit from the modeling methodology and analysis techniques.
• The data describes a generalized case and provides pathways to more complex modeling methods.

## 2 Background

Thermoset composites are notably used in different industries requiring high-performance materials. The polymer matrix is an amorphous material with low toughness which is usually supplemented with nanofillers. Nanofillers like GQDs have been shown to enhance the material properties of the matrix [1], [2], [3], [4]. Recent studies have affirmed these claims for GQD-epoxy composites [5]. To fully understand the inherent mechanisms responsible for property enhancements, this study uses molecular dynamics to model the polymer-GQD nanocomposite. Various interactions were observed, and mechanical simulations were performed to see the effect of GQDs on the properties. The data generated in this study was used to establish the findings linking GQD chemistry to the output mechanical properties. The simulated data includes the entire workflow of modelling the GQD-epoxy nanocomposite right from the inception of individual monomeric components.

## 3 Data Description

The simulation data is distributed in four main directories for the four modelled material systems – epoxy, GQD-epoxy, 4N-GQD-epoxy, and 6N-GQD-epoxy. Each directory has several sub-directories which include LAMMPS files for a specific simulation. Table 1 details the description of all the sub-directories and Table 2 details the description of the files within the sub-directories.

**Table 1:** Directory data description for the four material systems.

Directory name	Simulation Type	Simulation Details
step_1	Monomer generation	Generating individual monomeric components using the IFF-R forcefield
step_2	Bulk polymer at liquid density	Mixing the polymer components in a stoichiometric ratio and compressing the model to liquid density
step_3	Annealing	Inserting the GQD and cooling the model from elevated temperature to cure temperature
step_4	Crosslinking	Chemical crosslinking to form the polymer network
step_5	Annealing	Cooling down the model from cure temperature to room temperature and relaxing the model
step_6	Relaxation	Changing the forcefield from IFF-R to ReaxFF and equilibrating the model
step_7	Tension	Apply strain to the simulation box in three principal directions (x-, y-, and z- axis) separately

**Table 2:** File data description for the sub-directory contents.

File Types	File Details
dump.*	LAMMPS trajectory
*.data	LAMMPS structure
in.*	LAMMPS input script
*.log or log.*	LAMMPS log
*.moltemp	reaction template
*.ecoeffs	reaction template coefficients
*.txt	reaction map
*.csv	stress-strain data
*.reax	ReaxFF forcefield file

## 4 Experimental Design, Materials and Methods

The epoxy resin used in this study is the diglycidyl ether bisphenol F (DGEBF), sold commercially as EPON 862. The curing agent used in the diethyl toluene diamine (DETDA), sold commercially as Epikure W. The two molecules were modelled and mixed in a simulation box with the stoichiometric ratio of 2:1 using the IFF-R forcefield in LAMMPS software [6], [7], [8]. The selected stoichiometric ratio was selected to maximize the chemical crosslinking between the molecules during the virtual cure [9,10]. The mixture was multiplied to generate a larger model with the total atom count of 5616 atoms and the total molecule count of 144.

The initial density of the system was 0.09 – 0.10 g/cm^3^. Before densifying the system, the molecules were allowed to mix by ramping the system temperature down from 600 K to 300 K over 100 picoseconds (ps). A Nose-Hoover thermostat [11], [12], [13], [14] was used and the timestep was set to 1 femtosecond (fs). The simulation box was gradually densified by compression from all directions. The compression was simulated over 8 nanoseconds (ns) with the target density of 1.2 g/cm^3^.

**Fig. 1:**
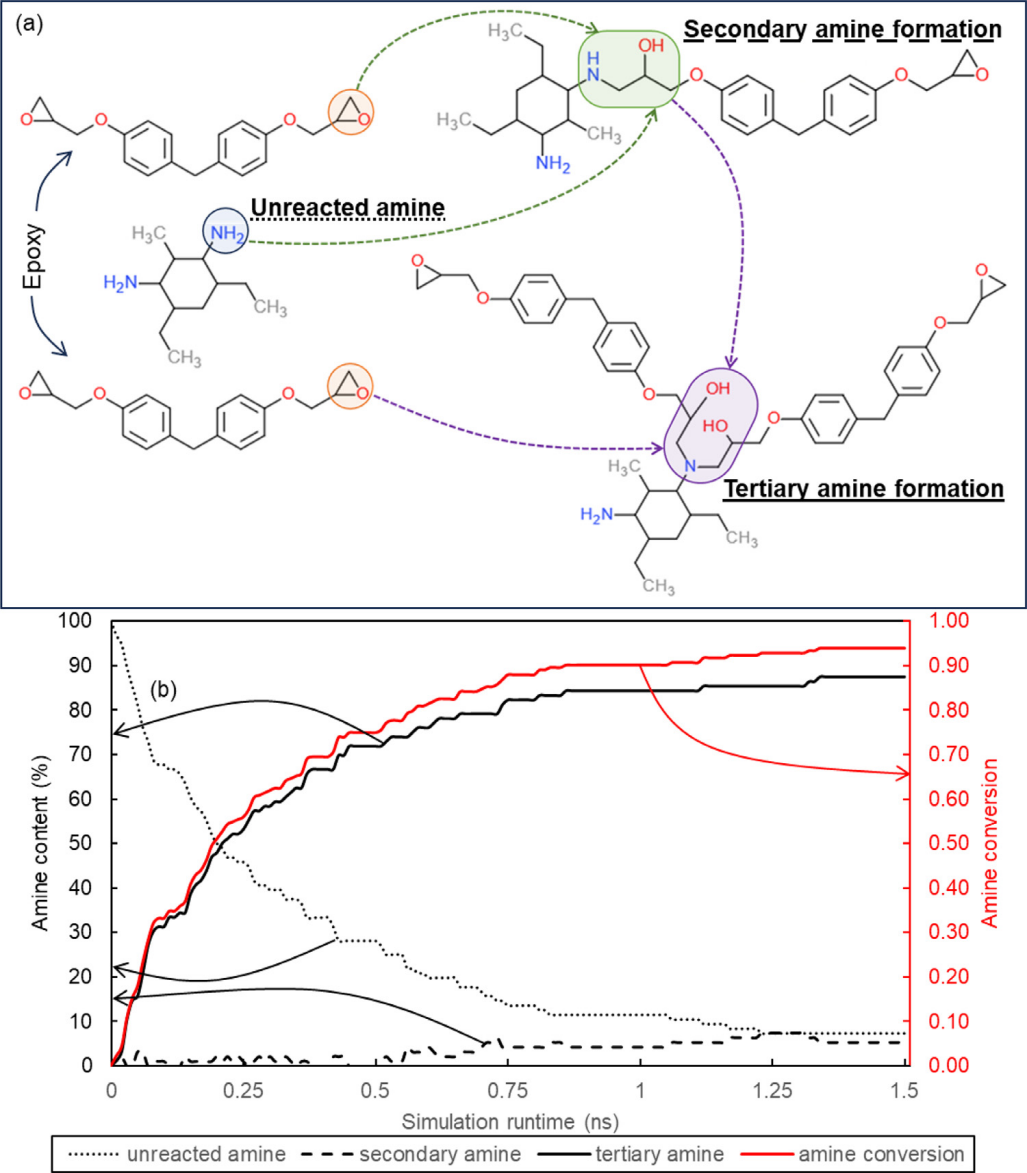
(a) Epoxy-amine reaction pathway for a bisphenol-F epoxy (DGEBF) and diethyl toluene diamine (DETDA) and (b)amine content and conversion during the chemical crosslinking simulation in a representative model with 90 % crosslink density.

**Fig. 2:**
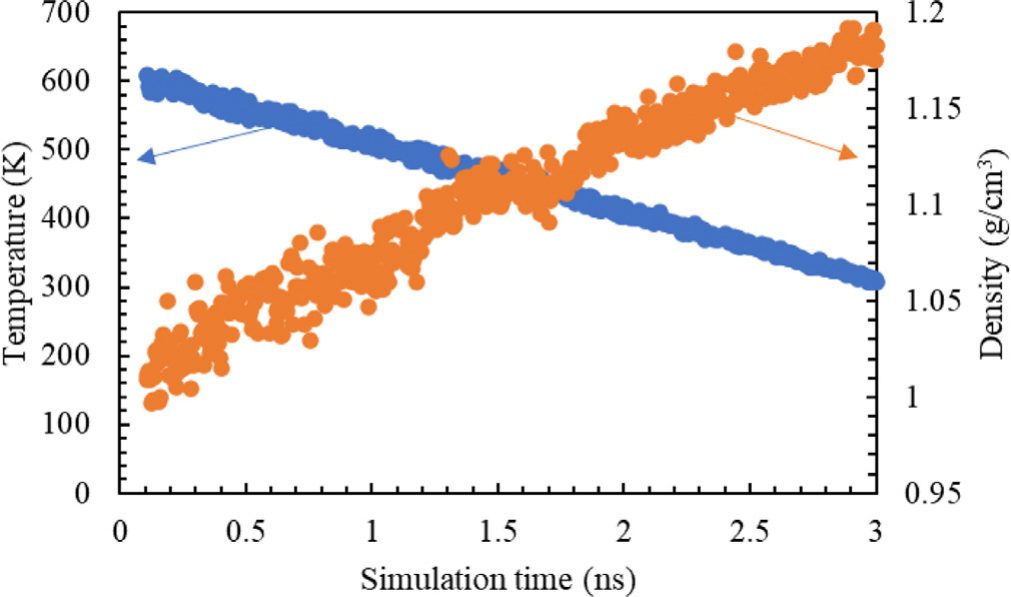
Temperature and density profile during annealing simulation.

**Fig. 3:**
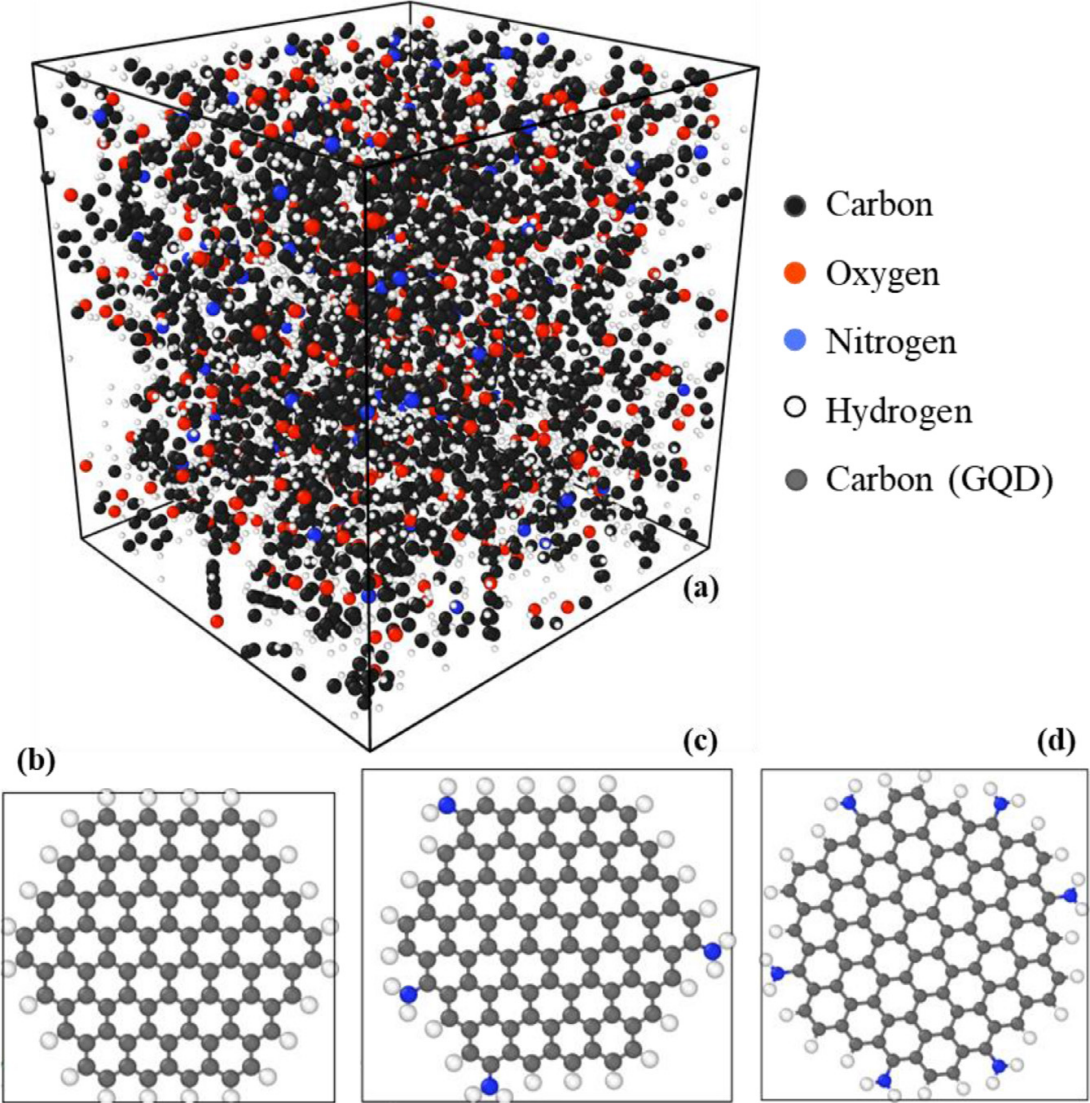
(a) Representative equilibrated epoxy model. (b) Isolated GQD model before integration (Top view), (c) Isolated 4N-GQD model before integration (Top view), and (c) Isolated 6N-GQD model before integration (Top view).

A virtual curing simulation was performed at 499 K over 1.5 ns using the REACTER tool within LAMMPS [15]. The simulated cure reaction is the two-step amine-epoxy reaction. Details on the curing simulation settings is described elsewhere [10]. To account for uncertainty of property prediction, five unique replicates were modelled. The average crosslink density between the five models was 81.77 ± 3.47%. Fig. 1 shows (a) the amine reaction and (b) amine content in a representative model and conversion during crosslinking simulations. Post-crosslinking, the models were annealed to promote optimal conformation of the newly formed network. The system temperature was ramped down from 600 K to 300 K with a constant cooling rate of 100 K/ns. Fig. 2 shows the temperature and density profile during the annealing simulation. Next, the system was equilibrated over 1 ns using a Nose-Hoover barostat and thermostat at 1 atm pressure and 300 K temperature respectively. Fig. 3(a) shows a representative MD model after full equilibration.

To perform the mechanical deformation simulations, the models were equilibrated by switching the forcefield parameters from IFF-R to ReaxFF [9,16]. This ReaxFF parameter set was chosen because of the accuracy of density prediction in various hydrocarbon-based materials [17]. As established by Odegard et al. [6], accurate density prediction is essential for capturing the corresponding mechanical response of the material. The equilibrated models were then deformed in the three directions (*x*-, *y*-, and *z*- direction). To allow the Poisson contraction, an NPT ensemble was used to relax the lateral directions. The maximum applied strain was set at 10% with the strain rate of 2 × 10^8^ /s and 0.1 fs timestep. The stress-strain data was then analysed to extract the Young's modulus and the yield strength of the material. A piecewise multi-linear curve fitting technique was used to extract the Young's modulus, Poisson's ratio and establish a yield point on the stress-strain curve.

A pristine GQD was modelled using an in-house Python script and LAMMPS. The edge carbon atoms were terminated with hydrogen atoms. The dimensions of the modelled GQD were 1.9 nm × 1.8 nm in zigzag configuration. Fig. 3(b) shows the GQD structure modelled in LAMMPS. The model contains 5616 epoxy atoms and 120 GQD atoms. The modelled GQD was combined with epoxy and annealed as demonstrated by Deshpande et al. [18]. The annealing simulation settings and rest of successive simulations were identical to the neat epoxy simulation described in previous section. Five replicates were modelled to account for uncertainty in property prediction.

The functionalized GQD was modelled using an in-house Python script and LAMMPS. The functional group of choice was the primary amine (NH_2_) and were randomly distributed on the edge of the GQD. Two f-GQD were modelled with different amount of amine groups. For the first sub-system (4N-GQD-epoxy), the total amount of functional groups accounted for 5.2% of the total mass of the GQD. The chosen structure is shown in Fig. 3(c), the structure has four primary amine groups and 2.1 × 1.9 nm in dimensions. For the second sub-system (6N-GQD-epoxy), the total amount of functional groups accounted for 7.6% of the total mass of the GQD. Fig. 3(d) shows the structure with six primary amine groups with dimensions 1.8 × 1.9 nm. As described in the previous section, the f-GQD and epoxy models were combined using LAMMPS and annealed. The model consisted of 5616 epoxy atoms and 128 f-GQD atoms. Post-annealing, the model was subjected to crosslinking. With the presence of the functional groups, a secondary chemical reaction was performed where the epoxy molecules reacted with the primary amines on the GQD. This secondary reaction is identical to the reaction occurring within the epoxy material due to similar chemistry.

## Limitations

Not applicable.

## Ethics Statement

The authors have read and follow the ethical requirements for publication in Data in Brief and confirm that the current work does not involve human subjects, animal experiments, or any data collected from social media platforms.

## CRediT authorship contribution statement

**Prathamesh P. Deshpande:** Conceptualization, Methodology, Software, Validation, Investigation, Resources, Data curation, Writing – original draft, Writing – review & editing, Visualization. **Ozgur Keles:** Conceptualization, Methodology, Investigation, Resources, Writing – review & editing, Supervision, Project administration, Funding acquisition.

## Data Availability

Molecular Dynamics Modeling of Graphene Quantum Dot Epoxy Nanocomposite To Predict Physio-Mechanical Properties (Original data) (Mendeley Data)
